# Culturally Responsive Pediatric Rehabilitation Interventions: A Scoping Review

**DOI:** 10.3390/bs16061031

**Published:** 2026-06-19

**Authors:** Ashley Albores, Annamarie Jump, Hana Rupnow, Cheyenne Schorlig, Patricia C. Coker-Bolt, Emerson Hart

**Affiliations:** The Graduate College of Health Sciences, Hawai‘i Pacific University, Honolulu, HI 96813, USAeehart@hpu.edu (E.H.)

**Keywords:** rehabilitation, cultural responsiveness, children, pediatrics, barriers, challenges, facilitators, limitations

## Abstract

Culturally responsive frameworks are essential for delivering equitable rehabilitation services to diverse communities. Culturally informed practices that use evidence-based strategies facilitate holistic, family-centered interventions. This scoping review explores the literature published over the last 5 years on barriers and facilitators to the use of culturally responsive interventions for children and families receiving pediatric rehabilitation services. Databases searched included PubMed, CINAHL Complete, Medline, Cochrane Library, and OTseeker. Search terms included cultural competence, culturally informed, culturally grounded, pediatrics, rehabilitation, physical therapy, occupational therapy, barriers, facilitators, and a combination of these terms. The review followed the Preferred Reporting Items for Systematic Reviews and Meta-Analyses (PRISMA) guidelines. Published intervention studies that identified the barriers and facilitators of culturally responsive care were included in this review. Data from presentations, non-peer-reviewed literature, published abstracts, and dissertations were excluded. Ten studies were included, two Level III, three Level IV, and five Level V, according to the commonly accepted research Levels of Evidence. The outcomes of these studies suggest that rehabilitation providers should consider how to implement tailored, culturally informed interventions to improve holistic, accessible care for all communities.

## 1. Introduction

Awareness and understanding of cultural responsiveness and humility, or lack thereof, can significantly impact the therapeutic relationship between rehabilitation providers and the children and families they serve. As providers engage with increasingly diverse populations, it is imperative that they adopt a culturally responsive lens across all aspects of care. This includes recognizing the influence of culture on engagement in meaningful activities and being aware of the barriers and facilitators to incorporating cultural considerations into therapeutic intervention plans. Building on the need for a dynamic and reflective approach to pediatric rehabilitation, it is important to consider how the terminology used to describe culturally informed practice has evolved over time.

Historically, the term “cultural competence” has been used to describe a practitioner’s ability to effectively engage with people from diverse cultural backgrounds to meet the social, cultural, and linguistic needs of individuals (CDC, 2023; U.S. Department of Health and Human Services, Office of Minority Health, 2018). It is important to acknowledge that the concept of cultural competence is criticized for implying that one can attain mastery in understanding, thereby overlooking aspects of culture rooted in human identity and lived experiences (Greene-Monton & Minkler, 2019). More appropriate terms include cultural responsiveness and cultural humility, which emphasize the ongoing process of learning and adapting in response to a client’s cultural identity, acknowledging and reflecting on potential personal biases, and committing to lifelong learning. Cultural responsiveness is the ability to actively recognize, respect, and adapt care, services, or interventions to align with the cultural values, beliefs, and needs of individuals and communities, emphasizing flexibility and ongoing responsiveness in practice (CDC, 2023). Cultural humility is a lifelong process of self-reflection and self-critique in which individuals recognize power imbalances, challenge assumptions, and engage in respectful partnerships with individuals from diverse backgrounds, rather than assuming mastery of a finite body of cultural knowledge (CDC, 2023; Fritter & Shihabuddin, 2024). Cultural humility is an ongoing process of self-reflection and lifelong learning that emphasizes awareness of power imbalances and respectful partnerships (Fritter & Shihabuddin, 2024). Due to the historical prevalence of the term “cultural competence,” this review will use that terminology only when reporting outcomes as described in the original studies. However, we will use “cultural responsiveness” and “cultural humility” interchangeably to reflect contemporary practices in rehabilitation publications.

Tailored, culturally responsive approaches are essential not only for the efficacy and effectiveness of rehabilitation services but also for enhancing accessibility, fostering patient–provider trust, and promoting positive experiences for clients and their families (Dumont et al., 2025). Providing culturally responsive care requires rehabilitation providers to maintain a level of awareness of potential personal and cultural biases that could impact interactions and the development of therapeutic relationships with patients from other cultural backgrounds (Banks & Carames-Foley, 2025). A recent study by Hailey et al. (2025) validates that culturally grounded, community-directed service models are necessary to ensure relevance, improved access, and family-centered care. Practitioners who are not trained in culturally responsive practices may not understand how to provide interventions reflective of Indigenous cultural values, language, or communal caregiving practices. In addition, when practitioners lack cultural awareness or fail to incorporate cultural humility, families may face provider bias, experience mistrust, stigma, or feel misunderstood—resulting in decreased engagement or withdrawal from care altogether, which can worsen health and developmental outcomes (Dumont et al., 2025; Shin et al., 2025).

Rehabilitation practitioners can adapt existing interventions to make them culturally relevant and support culturally informed, evidence-based practices (Dumont et al., 2025; Halsall et al., 2024). The Cultural Enhancement Model (CEM) is a framework designed to help practitioners adapt evidence-based practices (EBPs) to make them more culturally relevant and engaging for diverse communities (Walker & Bruns, 2013). While maintaining the core and essential components of an intervention, the model focuses on enhancing the way services are delivered to better reflect the cultural identities, communication styles, and preferences of the families being served. Areas are identified for enhancement based on therapists’ and clients’ local experience rather than a theoretically driven set of recommendations. The model outlines a five-phase process: (1) forming a community advisory team, (2) gathering information about cultural needs and service challenges, (3) developing enhancement strategies, (4) implementing staff training and practice changes, and (5) evaluating whether these adaptations may improve engagement and practitioner preparedness. The CEM was designed to help programs incorporate culturally relevant strategies into evidence-based practice to improve both community- and client-level engagement, and has been successfully used in social work and psychology practice. Although the CEM outlines strategies for delivering culturally responsive care within a structured framework, it has not been widely applied to pediatric rehabilitation interventions.

It is time for rehabilitation practitioners to acknowledge the shifting landscape of working with diverse families and children. Cultural responsiveness should not be viewed as optional, but rather as a critical lens through which barriers and facilitators to care are identified, understood, and addressed. What barriers and facilitators are reported in the literature regarding culturally responsive pediatric rehabilitation interventions for children and families, including educational and training approaches that support culturally responsive care? The aim of this scoping review is to explore the literature published over the last 5 years on barriers and facilitators to the use of culturally responsive interventions for children and families receiving pediatric rehabilitation services. The purpose of this review is to capture up-to-date evidence on culturally responsive care, highlighting contemporary approaches and advancements. The findings from this review will inform more equitable, family-centered, and responsive service delivery across diverse pediatric populations.

## 2. Materials and Method

The scoping review adhered to the Preferred Reporting Items for Scoping Reviews (PRISMA-Scoping Review [ScR]) guidelines and incorporated recommended processes for conducting a scoping review. The guiding research question for this scoping review was: What are the barriers and facilitators to culturally responsive pediatric rehabilitation interventions?

### 2.1. Information Sources and Search Strategies

A comprehensive literature search was conducted from 12 May 2025 to 31 October 2025 to ensure all relevant research was included. A search for relevant literature was conducted using electronic databases, including PubMed, CINAHL Complete, Medline, the Cochrane Library, Scopus, Google Scholar, and OTseeker. These databases encompass a broad range of relevant studies and also feature field-specific sources to ensure the inclusion of significant research. Search terms included cultural competence, culturally informed, culturally grounded, culturally responsive, pediatrics, rehabilitation, physical therapy, occupational therapy, barriers, facilitators, and combinations of these terms. An example of full electronic search strategy used for this review is (“cultural competence” OR “cultural humility” OR “cultural responsiveness” OR “culturally grounded”) and (“occupational therapy” “physical (physio)therapy” OR “occupational therapist” OR “physical (physio)therapist” “OT” OR “OT or PT practitioner”) AND (intervention* OR rehabilitation* OR treatment* OR training* OR therapy* OR service*).

### 2.2. Eligibility Criteria

The inclusion and exclusion criteria were designed to ensure that the literature review aligned with the aim of the scoping review, demonstrated methodological rigor, and represented current knowledge in this field. The included studies were limited to peer-reviewed research focused on culturally responsive intervention practices. To meet inclusion criteria, studies were required to clearly describe how care, services, or approaches can be intentionally designed to recognize, respect, and adapt to the cultural values, beliefs, and needs of individuals and communities (CDC, 2023). The studies were required to be published in English and dated between 2020 and 2025. Exclusion criteria: studies published prior to 2020 and studies that did not meet the inclusion criteria, including systematic reviews, scoping reviews, dissertations, and conference presentations.

### 2.3. Study Selection

**Figure 1:**
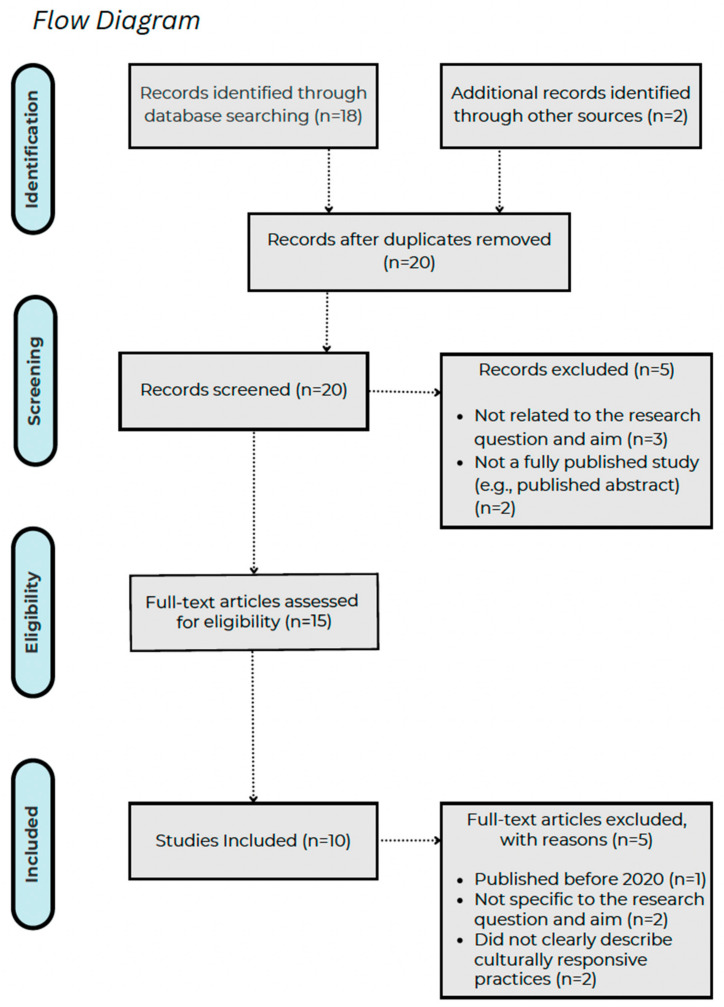
PRISMA Flow Diagram.

The study selection process followed the PRISMA-ScR (Tricco et al., 2018) guidelines and was conducted in several phases. First, relevant literature was retrieved from selected databases using the eligibility criteria outlined in the previous section. In the second phase, the 20 retrieved records were manually screened by title and abstract by two authors, and the authors reached consensus that articles were excluded at this stage if they did not meet the eligibility criteria, were not a fully published study (e.g., published abstract), were not specific to pediatric rehabilitation, did not provide sufficient details about the study methods, or did not embody culturally responsive intervention practices. After screening, the final phase involved the same two reviewers examining the full-text articles to confirm their eligibility for inclusion in the data extraction phase. Only empirical studies that met all eligibility criteria upon full-text review were retained. A detailed overview of the study selection process is illustrated in the PRISMA flow diagram (see Figure 1). The search initially identified 20 articles related to the aims of this scoping review. Two authors conducted full-text evaluations of the 15 selected studies, assessed their quality, extracted relevant data, and excluded 5 articles. The final inclusion of 10 articles was determined by consensus, ensuring that all articles met the established eligibility criteria (see Figure 1).

### 2.4. Study Extraction and Charting

Data were systematically extracted from the 10 included studies using a deductive thematic approach, focusing on the research question. The data extraction process involved labeling the information according to predefined themes and summarizing the results in a structured form. One author was responsible for extracting the data, while the other authors reviewed the process and results. In case of any discrepancies, the authors collaboratively resolved the issues to ensure consistency in the final outcome. Information on the characteristics of selected studies, including the author(s) and year of publication, participant age, sample size, study type, intervention, and outcome measures, is presented in Table 1. A risk-of-bias assessment was conducted to identify any potential biases in the results or conclusions of each study (Table 2).

## 3. Results

The aim of this scoping review was to explore the literature published over the last 5 years on barriers and facilitators to the use of culturally responsive interventions for children and families receiving pediatric rehabilitation services. Ten studies met the inclusion criteria and provided relevant information on barriers and facilitators to providing culturally responsive pediatric rehabilitation interventions. The information from these articles was divided into two themes: barriers and facilitators.

### 3.1. Barriers to Use of Culturally Responsive Interventions

Five of the ten included studies discussed barriers related to cultural responsiveness in pediatric occupational therapy intervention. One of these studies was a Level III study, one a Level IV study, and three were Level V studies (Table 3). All studies provided evidence that barriers to cultural sensitivity and competence impact the effectiveness and potential benefits of service interventions (Table 3).

Across the included studies, consistent patterns emerge demonstrating that cultural mismatches between families and therapeutic services act as significant barriers to receiving culturally responsive pediatric intervention, particularly for children with autism and related developmental conditions included in these studies. The qualitative and mixed-methods studies, which often centered on immigrant or ethnically diverse populations, consistently report challenges such as language barriers, acculturation differences, limited provider cultural humility, and discomfort navigating or advocating within the current Western medical models. These studies emphasize relational and contextual factors, highlighting how family experiences, trust, and cultural beliefs shape service engagement. In contrast, intervention-focused studies examining culturally adapted programs (e.g., Parents Taking Action, Cog-Fun adaptations) provide preliminary evidence that tailoring intervention content, delivery methods, and communication strategies to align with cultural values and family routines can improve engagement and perceived relevance.

However, important inconsistencies are evident across the study designs and populations that met the inclusion criteria for this review. Qualitative studies primarily emphasize family and caregiver perspectives, frequently highlighting systemic inequities and unmet needs within service delivery. The intervention studies are small-scale, pilot-only, and focus more narrowly on feasibility and acceptability, with limited examination of long-term outcomes. Additionally, many studies rely on single-stakeholder perspectives (caregivers or providers), resulting in fragmented understandings of culturally responsive care. Variability across cultural groups (e.g., Chinese immigrants, Latinx, Black Americans, Sri Lankan Tamils) and service contexts (e.g., early intervention, perinatal care, pediatric rehabilitation) further limits cross-study comparability and generalizability. Across both descriptive and intervention studies, small sample sizes, lack of control groups, and potential social desirability bias constrain the strength of the evidence. Despite these limitations, these studies highlight that a clear pattern exists in which culturally responsive care is most effective when it integrates cultural humility, family-centered approaches, and context-specific adaptations co-developed with communities. Collectively, the findings underscore the need for more rigorous, multi-stakeholder, and longitudinal research to better understand how culturally responsive interventions can be systematically implemented and sustained across diverse populations and pediatric rehabilitation settings.

### 3.2. Facilitators to Use of Culturally Responsive Interventions

Five of the ten included studies discussed facilitators to cultural responsiveness in pediatric therapy intervention. One study was Level III, one Level IV, and three Level V (Table 4). All studies provided evidence that cultural awareness, individualized care, and continuous training is effective and potentially beneficial when used during service interventions (Table 4).

Across the included studies, the evidence suggests that culturally responsive training, intervention design, and community collaboration are key facilitators of more equitable and effective therapy practice. However, patterns vary across study designs and contexts. Qualitative and mixed-methods studies were conducted with diverse ethnic minority families and emphasized experiential outcomes, demonstrating how culturally adapted interventions co-developed with families, cultural experts, and community stakeholders improve engagement, trust, and perceived relevance. These studies frequently highlight relational and contextual facilitators, such as shared cultural understanding, respect for family preferences, and flexibility in service delivery. In contrast, the intervention studies, typically conducted with providers, focused on outcomes related to increased cultural awareness, knowledge, and perceived preparedness, but provide limited evidence of how increased knowledge would translate into more culturally responsive practice.

Inconsistencies emerge across populations and settings in the included articles. Studies involving specific cultural groups (e.g., immigrant families versus historically marginalized U.S. populations) identify distinct primary barriers, such as language access and acculturation challenges, as well as systemic inequities and mistrust, suggesting that culturally responsive approaches must be tailored to context. Additionally, while some intervention studies report improved engagement and satisfaction, few measure functional or long-term outcomes, limiting conclusions about effectiveness. Variability in intervention type, delivery setting (e.g., early intervention, community-based care, educational programs), and stakeholder involvement further complicates cross-study comparisons. Despite these differences, studies note key facilitators to culturally responsive care, including workforce diversity, individualized and flexible service delivery, and supportive system-level policies for underrepresented populations. However, the predominance of small, self-selected samples, lack of control groups, and reliance on short-term or subjective outcomes underscore the need for more rigorous, multi-site, and longitudinal research to strengthen the evidence and guide scalable implementation of culturally responsive therapy interventions.

## 4. Discussion

The results of this scoping review suggest that advancing culturally responsive pediatric rehabilitation may be supported by training practitioners in cultural awareness and humility, using a structured framework to guide the cultural adaptation of interventions, and engaging practitioners and communities who share cultural backgrounds with families to enhance relevance, trust, and effectiveness of care (Banks & Carames-Foley, 2025; Dumont et al., 2025; Halsall et al., 2024; Xu et al., 2023; Yu et al., 2025). The majority of included studies focused on interventions involving children, families, and professionals, while some studies examined practitioner training as a mechanism to enhance culturally responsive care delivery in pediatric rehabilitation settings. These educational interventions exemplify systems- and workforce-level facilitators that support the implementation of culturally responsive pediatric rehabilitation interventions for children and families. Across the included studies and broader evidence from studies on culturally sensitive pediatric primary care (Okoniewski et al., 2022), findings indicate that culturally responsive interventions are both feasible and acceptable, with emerging evidence of the effectiveness of tailored interventions to improve family engagement and, in some cases, health outcomes.

These findings underscore that barriers to culturally responsive practice extend beyond individual communication challenges to reflect broader gaps in training, workforce diversity, and openness to engaging with different cultures in clinical care. Language differences and a lack of shared cultural experiences can hinder the provision of holistic, family-centered care, especially when practitioners lack structured approaches to effectively incorporate cultural values into intervention planning (Lim & Nakamura, 2022). For example, it is important to consider that cultural beliefs about disability and child rearing shape family expectations, engagement, and participation in therapy, underscoring the need to position culture as a central client factor in pediatric rehabilitation (Grandpierre et al., 2018; Hailey et al., 2025; Lim & Nakamura, 2022; Yu et al., 2025).

**Figure 2:**
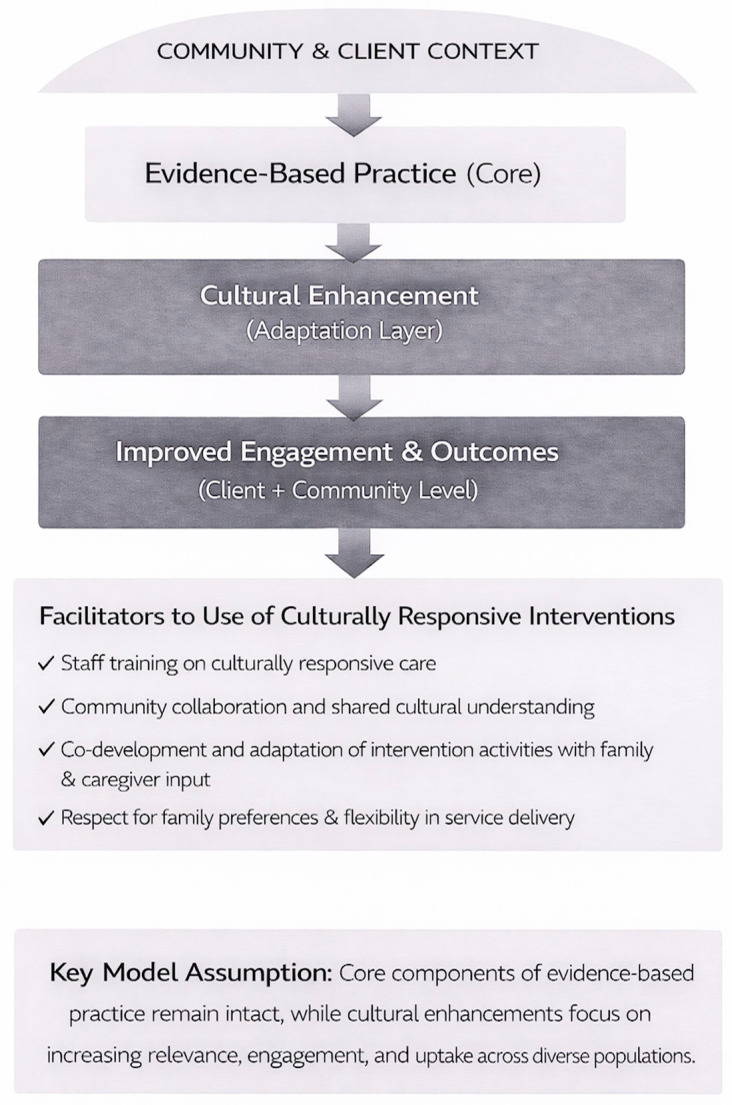
Flowchart of the Integration of Cultural Enhancement Model With Culturally Responsive Practice.

Addressing these challenges requires a more intentional and sustained focus on cultural responsiveness across professional preparation and practice. Enhancing cultural responsiveness among rehabilitation practitioners requires commitment at both the individual and systems levels. The CEM proposes a strategy to address both community- and client-level engagement within a framework that can be flexibly and locally applied to facilitate the adaptation and development of culturally responsive interventions (Walker & Bruns, 2013) (Figure 2). Using this model, practitioners can integrate culturally aligned communication approaches, family roles, and community resources, while adapting interventions to reflect families’ language, perceptions of disability, and expectations for child development. The goal is to improve trust, access, and participation, particularly among groups who may distrust or feel alienated by traditional clinical services. The CEM demonstrates that culturally responsive interventions can be integrated into EBPs in practical, feasible ways without weakening the evidence base, helping bridge the gap between research-driven models and real-world, culturally diverse care settings (Figure 2).

## 5. Implications for Practice

The findings from this scoping review suggest that promoting culturally responsive care involves more than surface-level adjustments. Providers should prioritize linguistic accessibility (e.g., offering interpretation services and translating educational materials), integrate family and community values into intervention planning, and acknowledge power dynamics in clinical encounters. Collaboration with community health workers or cultural liaisons could further strengthen engagement, trust, and continuity of care. In addition, workforce diversification efforts may improve cultural alignment between practitioners and the communities they serve.

Integrating culturally responsive frameworks, such as the CEM, into entry-level education and continuing education may support practitioners in developing the skills and confidence needed to provide culturally attuned, family-centered care (Banks & Carames-Foley, 2025; Fan & Chen, 2024). Rehabilitation educational programs can include culturally responsive coursework for future practitioners and perpetuate integration of cultural knowledge in continuing education courses (Banks & Carames-Foley, 2025; Fan & Chen, 2024).

Most importantly, this scoping review highlights gaps in the current evidence base on culturally responsive practice. Future research should examine the long-term outcomes of culturally adapted interventions, include more diverse participant populations, and employ research designs accessible to families across linguistic and socioeconomic contexts. Expanding community-engaged and participatory action research methods may further help ensure that the lived experiences of the communities guide intervention development. Ongoing reflection, relationship-building, and advocacy at the practitioner, organizational, and educational levels are necessary to ensure that cultural responsiveness evolves from intention into sustained, real-world practice. By committing to this work, the profession can help reduce disparities and promote meaningful participation for all children and families.

## 6. Conclusions

This scoping review demonstrates that culturally responsive approaches may strengthen the effectiveness and relevance of pediatric rehabilitation interventions; however, more rigorously designed studies are needed to better establish their impact on rehabilitation outcomes. When practitioners actively honor family values, communication norms, and culturally situated understandings of child development, intervention strategies are more likely to be accepted, integrated, and sustained in daily routines. These findings reinforce that cultural responsiveness is not an optional enhancement, but should become a core component of ethical, client-centered care.

## 7. Limitations

The strengths of this scoping review include consistency with PRISMA guidelines/flow diagram for reporting and guiding article search results. Limitations of this review include its specific focus on the recent literature published between 2020 and 2025, which aimed to capture the latest trends and avoid duplication of previous work on culturally responsive rehabilitation interventions. This scoping review included only 10 studies, most of which were classified as Level V evidence, warranting caution in interpretation of the results due to the limited strength and rigor of the available evidence. During the scoping review process, the search criteria for “pediatric or child or children or infant or adolescent” may have excluded relevant studies utilizing adults or subjects of all ages. Given the emphasis on the pediatric population, it is possible that relevant studies on culturally responsive interventions for other ages were excluded. This review included only English-language studies, which may introduce language bias, and may also be subject to publication bias due to the exclusion of unpublished or non-indexed work. Additionally, the heterogeneity of the included studies in terms of research design, populations, and interventions limits the comparability across findings. Many studies also had small sample sizes and lacked control groups, which may reduce the strength and generalizability of the findings.

## Figures and Tables

**Table 1:** Evidence Table for Included Studies.

Evidence Table					
Author/Year	Level of Evidence Study Design Risk of Bias	Participants Inclusion Criteria Study Setting	Intervention and Control Groups	Outcome Measures	Results
		Participants:		Cultural Competence Self-Assessment Checklist–Revised (CCSACR) (Central Vancouver Island Multicultural Society, 2004) administered pre- and post- intervention as well as a qualitative questionnaire on self-perceived changes	A brief, curriculum-based educational module significantly improved occupational therapy students’ self-reported cultural awareness, skills, and preparedness to provide culturally effective care for Hispanic families. Significant improvements were found in cultural awareness (*p* = 0.013) and cultural skills (*p* = 0.038) and self-perceived improvements in awareness, knowledge and preparedness for culturally effective practice.
	LOE: Level IV Study Design:	30 occupational therapy mater’s students, 28 female, 2 male	Intervention: 2 h cognitive–behavioral-based		
	Mixed Methods,	Inclusion Criteria:	intervention		
(Banks & Carames-Foley, 2025)	pre–post	occupational therapy	module on		
	interventional	master’s students enrolled	culturally		
	study	in a pediatric course	effective care for		
	ROB: M	Study Setting: an entry-level occupational	Hispanic families		
		therapy master’s program			
(Dumont et al., 2025)	LOE: Level V Study Design: Qualitative study w/focus groups and interviews ROB: Qualitative Study	Participants, 12: 4 parents/caregivers, 2 cultural experts, 6 OTPs Inclusion Criteria: Of Black American [BA] ethnicity and/or directly work/care for with BA Autistic children and/or culture Study Setting: Virtual focus groups and interviews via Zoom platform	A caregiver-mediated, sensory–behavioral approach Using Sensory Integration [OT-SI] framework and related caregiver coaching models	Brief description of method and process of analysis: Interview and focus group discussions Key Themes relevant: BA Families of Autistic Children report facing many challenges, biases, and lack of support in receiving therapy.	Five Barrier themes emerged: Access and delivery of services, Systemic barriers, Lack of cultural humility practices, scheduling, and Obtaining an autism diagnosis. Seven Supportive Themes Emerged: Access to additional resources, Improved cultural humility and client-centered practices, Improved use to therapeutic principles, Parent and caregiver preferences and support building, Changes at legislative and professional levels, Increased Autism Education/Training, Improved Therapeutic service delivery
(Fan & Chen, 2024)	LOE: Level V Study Design: pilot, single-case, qualitative semi-structured interviews with parents, grandparents, as well as service providers	Participants: one Chinese Canadian family receiving intervention services for 2 children with ASD, set in Canada	A pilot single case study using a modified standard parent-mediated, clinic-based therapeutic approaches to better align with the family’s cultural values, beliefs, and caregiving practices.	Methods: Exploratory case study involving 6 stages; pilot investigation, single case study design Key themes: “three major themes in the form of tensions: (1) tensions within the family; (2) tensions within the therapeutic relationship, and (3) tensions when addressing culture in therapy”	Many internal and external tensions were found between family members and therapeutic service providers, especially in relation to cultural differences in goal setting. All agreed culture is important—but there was a lack of willingness to discuss the cultural issues within ASD services. Even though service providers agreed cultural influence is important, results suggested they were not providing adequate culturally relevant and family centered care.
(Golos et al., 2021)	LOE: Level IV Study Design: Mixed-method questionnaires ROB: M	Participants: 28 OTPs Inclusion Criteria: certified to use Cog-Fun intervention protocol for children 5 to 10 and experienced with the protocol for children from the UltraOrthodox (UO) Community.	Cog-Fun Interventions with UO Children with ADHD.	30-min questionnaire that examine the UO participant perception, professional and personal experience, and cultural relevance	Themes included: parental knowledge regarding ADHD diagnosis and intervention, parental perceptions and attitudes regarding ADHD diagnosis and medication, factors affecting communication between the OTP, parents, and teachers It is important to adapt intervention protocols to habits, routines, and lifestyle of UO families. It is important for therapist to have knowledge of the UO routines, family structure, religion and more to provide effective therapy using the Cog-Fun interventions.
(Halsall et al., 2024)	LOE: Level V Study Design: Qualitative study using non-probability sampling and interview for data collection ROB: Qualitative study where a small sample was explored from a specific population where resources and time were limited	Participants: 8 total, recruited via social media platform, X. Inclusion: respondents invited to participate if registered with UK regulatory body (Health and Care Professions Council); members of their professional body (Royal College of Occupational Therapists); and employed by NHS and working in an NHS community perinatal mental health service	Qualitative interviews with practitioners who described how they adapt their approaches in practice to better meet the cultural, social, and contextual needs of these populations.	8 introductory questions to collect participant demographics and info about employment and training 8 open-ended questions about participants’ experiences working with mothers from ethnic minorities, enablers and barriers that affect treatment, and the support they believed was required to improve their practice	Occupational therapists perceive multiple cultural and systemic barriers when supporting perinatal mental health needs of mothers from ethnic minority backgrounds. Key barriers included cultural stigma surrounding mental illness, fear or shame related to seeking help, language differences, and challenges associated with using interpreters, all of which were believed to reduce access to and engagement with services. Practitioners also noted that limited cultural understanding and lack of workforce diversity could hinder effective care.
(Rakic et al., 2022)	LOE: Level III Study Design: Cross-sectional study ROB: Social desirability minimized by anonymity. 7 of 9 Swiss Pediatric Oncology Group (SPOG) stations participated, however largest SPOG station did not participate which could represent bias	Participants: All Swiss pediatric oncology care providers caring for pediatric (0–18 years) cancer patients. Included all occupational groups in direct contact with patients (e.g., nurses, physicians, psycho-oncologists, social workers, or rehabilitation specialists such, as physiotherapists) at 7 SPOG stations	Examination of healthcare providers’ self-reported cross-cultural competencies, experiences, and perceived challenges in delivering care to culturally diverse pediatric oncology populations.	Cross-Cultural Competence of Healthcare Professionals (CCCHP) questionnaire, 27 items	There were notable variation in cross-cultural competence among pediatric oncology care providers in Switzerland, with differences observed across professional disciplines. Social workers demonstrated the highest levels of cross-cultural competence, while physicians and social workers reported more positive attitudes toward culturally diverse care compared to nurses. Commonly identified barriers to cross-cultural care included language differences, differing cultural values, and varying understandings of illness among families. Key facilitators include access to formal training in cross-cultural communication and care, the availability and effective use of professional interpreters and language support services.
(Shanmugarajah et al., 2022)	LOE: Level V Study Design: Qualitative study with semi-structured interviews and content analysis ROB: Small sample size used in qualitative study. Language barriers due to questions being asked in English (not native language)	Participants: 8 mothers of children with ASD who immigrated to Canada from Sri Lanka. All children were 16–23 yo Inclusion criteria: parents of children diagnosed with ASD, identified as an immigrant or first-generation Canadian from South Asian region, and people who could communicate in English with interviewer Setting: private room at Autism Centre	Examination of how cultural beliefs, immigration experiences, and systemic factors shape mothers’ understanding of autism and their engagement with services.	In-person interviews from 30–60 min each. Interviews were transcribed and coded using rigorous analysis to create meaning units including the creation of themes: facilitators and barriers for occupational therapy interventions within the Canadian Healthcare system.	The study found that mothers’ understandings of autism were strongly influenced by cultural beliefs and family expectations, which often differed from Western biomedical perspectives. Many mothers underreported their cultural needs out of fear of being misunderstood or dismissed, contributing to reduced collaboration in intervention planning. The findings underscore the importance of cultural humility, improved communication supports, and family-centered approaches in occupational therapy and related services to better meet the needs of immigrant families supporting children with autism.
(Terol et al., 2024)	LOE: Level IV Study Design: Qualitative study w/questionnaires, focus groups, and interviews ROB: Qualitative Study	Participants: 13 caregivers, 6 autistic individuals, and 9 professionals Inclusion Criteria: caregivers of individuals with autism, professionals who work with young children (0–8 years old) with autism, or self-identified autistic individuals, must be 18 years of age or older, and must live in Paraguay Setting: Paraguay	Cultural adaptation of a caregiver-mediated intervention for families of young autistic children, guided by input from community members.	Methods: Online demographic questionnaires were completed in Spanish. Focus groups and interviews were held over zoom in Spanish. All groups and interviews were recorded and transcribed in Spanish, then translated to English by one of the authors and verified by a separate author. Summaries were generated and sent to participants to review and confirm, clarify, or make modifications.	Community-informed cultural adaptation substantially enhanced the relevance and acceptability of the Parents Taking Action (PTA) intervention for caregivers of young autistic children in Paraguay. Caregivers, service providers, and autistic individuals identified the need for adaptations at both the surface and deep-structure levels, including the use of locally familiar language and examples, removal of U.S.-specific references, greater flexibility in delivery format, and expanded content addressing sensory needs, play development, and caregiver emotional support.
(Xu et al., 2023)	LOE: Level V Study Design: Qualitative study with interviews or focus group discussions with stakeholders (snowball sampling)	Participants: parents and/or primary caregiver of children or adults with ASD, the child under care is over 10 years old, identifying as Chinese and was foreign-born, and fluent in Mandarin or Cantonese, set in the United States	Cultural adaptation of a parent psychoeducational intervention designed for Chinese immigrant families of young children with autism spectrum disorder (ASD).	Methods: Interviews and focus groups with families following Parents Taking Action (PTA) intervention	Few interventions are being adapted to be culturally responsive despite the need for adaptation to meet the needs of diverse populations. Clear structure of how to gather stakeholder input and synthesize the process of coding and cultural adaptation of a parent training intervention. Future intervention research should test and modify the cultural adaptation coding system as different communities may have unique adaptation needs.
(Yu et al., 2025)	LOE: Level III Study Design: Mixed methods, single group, pre–post pilot study ROB: time constraints of intervention efficacy, small sample size, self-selected (highly motivated), self-reported assessments	Participants: 30 caregivers and their children with IDD Inclusion criteria: adult (18+) female caregiver self-identified as Latino/a/x or of Latin American decent and had a child with IDD Setting: recording of telephone or video chat for pre–post test. Interventions given virtually individually. Group sessions were planned 3 in-person, but switched to virtual after 2 sessions.	10 individual virtual sessions and 3 group sessions led by trained promotoras for education about nutrition, physical activity, stress management, and self-efficacy.	Psychosocial Outcomes: Social Support: MSPSS Health Related Self-Efficacy: SRAHP Home Environment: ISCOLE Behavioral Outcomes: Diet: DSQ Physical Activity: CHAMPS Sleep Disturbances: Pittsburgh Sleep Quality Index Screen Time Health Outcomes: BMI Family Caregiver Depression and Stress: CESD-10 Quality of Life: PROMIS-10	A culturally tailored, community-based health promotion program delivered by trained Promotoras, was effective in improving psychosocial, behavioral, and health-related outcomes among Latino families of children with intellectual and developmental disabilities. Families demonstrated significant gains in caregiver well-being, stress management, self-efficacy, health knowledge, and healthy behaviors related to nutrition and physical activity following participation. The Promotora model, which emphasized shared cultural background, trust, and community connection, enhanced engagement and relevance of the intervention.

Note. ASD = Autism Spectrum Disorder; BA = Black American; CESD-10 = Center for Epidemiological Studies Depression Scale; CHAMPS = Community Health Activities Model Program; IDD = Intellectual Developmental Disability; ISCOLE = International Study of Childhood Obesity, Lifestyle and the Environment; LOE = Level of Evidence; MSPSS = Scale of Perceived Social Support; N/A = Not Applicable; OT = Occupational Therapy; OTP = Occupational Therapy Practitioner PROMIS-10 = Patient-Reported Outcomes Measurement Information System; PTA = Parents Taking Action; ROB = Risk of Bias; SRAHP = Self-Rated Abilities for Health Practices; UO = Ultraorthodox.

**Table 2:** Risk-of-Bias Table for Selected Studies.

Risk of Bias for Before–After (Pre–Post) Studies with No Control Group (One Group Design)												
Citation	Study Question or Objective Clear	Eligibility or Selection Criteria Clearly Described	Participants Representative of Real-World Patients	All Eligible Participants Enrolled	Sample Size Appropriate for Confidence in Findings	Intervention Clearly Described and Delivered Consistently	Outcome Measures Pre-Specified, Defined, Valid/Reliable, and Assessed Consistently	Assessors Blinded to Participant Exposure to Intervention	Loss to Follow-Up After Baseline 20% or Less	Statistical Methods Examine Changes in Outcome Measures from Before to After Intervention	Outcome Measures Were Collected Multiple Times Before and After Intervention	Overall Risk of Bias Assessment (Low, Moderate, High Risk)
(Banks & Carames-Foley, 2025)	Y	Y	Y	Y	Y	N	Y	N	NR	Y	N	M
(Golos et al., 2021)	Y	Y	Y	Y	Y	N	Y	N	NR	N	N	M
(Terol et al., 2024)	Y	Y	Y	Y	Y	N	Y	N	NR	N	N	M
(Yu et al., 2025)	Y	Y	Y	NR	NR	Y	Y	N	NR	Y	N	L

Note. Y = yes; N = no; NR = not reported. Scoring for overall risk of bias assessment is as follows: 0–3 N, Low risk of bias (L); 4–8 N, Moderate risk of bias (M); 9–11 N, High risk of bias (H). Citation. Table format adapted from National Heart, Lung, and Blood Institute (2014). Quality assessment tool for before–after (pre–post) studies with no control group. Retrieved on 20 May 2025 from https://www.nhlbi.nih.gov/health-topics/study-quality-assessment-tools.

**Table 3:** Barriers to Use of Culturally Responsive Interventions.

Study (Year)	Design & Evidence Level	Population/Sample	Key Barriers Identified	Implications
Fan and Chen (2024)	Qualitative, single-case pilot study Level V	One Chinese family (grandparents, parents, and two sons with ASD) immigrants and three service providers in Canada.	Lack of collaboration between providers and families for goal-setting and services.	Providers benefit from training in culturally responsive communication and collaborative goal setting. Future studies should explore cultural sensitivity with larger sample sizes and diverse perspectives.
Golos et al. (2021)	Mixed methods questionnaire Level IV	28 Pediatric occupational therapists treating ADHD in the UO community in Israel.	Parental knowledge and perceptions of ADHD, communication, and adapting intervention protocols to the lifestyle of UO families.	Providers should expand knowledge acquisition, knowledge transfer, and application of knowledge for interventions with diverse populations.
Rakic et al. (2022)	Cross-sectional national survey Level III	183 Pediatric oncology care providers (OTs, nurses, physicians, social workers, PTs) in Switzerland.	Language barriers, differing cultural values, varying levels of illness understanding across families additionally variability of cross-cultural competence across providers.	Profession-specific cultural competence training is beneficial and can increase use of professional interpreters, and incorporation of cultural mediators to improve equitable care.
Shanmugarajah et al. (2022)	Qualitative study with semi-structured interviews Level V	Eight Sri Lankan Tamil immigrant mothers of children with ASD in Canada.	Mothers underreporting cultural practices due to fear of misunderstandings and language barriers in healthcare and school settings.	Institutional support is needed to enhance provider communication skills to foster cultural safety. This should include inviting families to share cultural values during intervention planning.
Xu et al. (2023)	Qualitative study with interviews and focus groups Level V	Six Chinese immigrant parents of children with ASD and six providers in a US Midwestern city.	Language barriers, lack of resources in native languages, and cultural norms discouraging disagreement with providers.	Multilingual resources are important to support provider awareness of cultural power dynamics to reduce disparities in care.

**Table 4:** Facilitator to Use of Culturally Responsive Interventions.

Study (Year)	Design & Evidence Level	Population/Sample	Key Facilitators Identified	Implications
Banks and Carames-Foley (2025)	Mixed-methods pre–post interventional study Level IV	30 occupational therapy master’s students enrolled in a pediatric course in New York City	A 2-h cognitive–behavioral-based intervention module on culturally effective care for Hispanic families demonstrated self-reported improvement of cultural awareness, skills and readiness for culturally effective care.	Early workforce training may effectively create culturally responsive, family-centered practices.
Dumont et al. (2025)	Qualitative study with focus groups and interviews Level V	Four parents and caregivers of Black American autistic children, six occupational therapists, and two cultural experts in the United States	Access to additional resources, improved cultural humility and client-centered practices, improved use of therapeutic principles, parent and caregiver preferences and support building, changes at legislative and professional levels, increased autism/training, and improved service delivery access.	Community collaboration supports cultural adaptation of interventions to improve local relevance, community trust, and service accessibility.
Halsall et al. (2024)	Qualitative study with semi-structured interviews Level V	Eight occupational therapists providing care for ethnic minority perinatal mothers in the United Kingdom	Increase diversity of occupational therapy workforce and foster greater cultural sensitivity in practice to improve quality of interventions to address themes of cultural barriers, personal trauma or shame of mental illness, and the experience of using interpreters.	There is a need for practitioner self-reflection on cultural identity and advocacy for workforce diversity to provide culturally responsive mental health interventions.
Terol et al. (2024)	Qualitative study with semi-structured interviews Level IV	28 caregivers, autistic individuals, and professionals in Paraguay	Adapting language in materials to local nomenclature and concepts, inter-professional teams of healthcare providers and caregivers to lead trainings, making content accessible with local resources, hybrid delivery models, use local government metrics for milestone tracking, providing space for social supports through storytelling, and adjusting interventions or advocacy depending on regional government systems.	Interventions can be adapted through community-led processes to improve acceptability, relevance, and sustainability of pediatric interventions.
Yu et al. (2025)	Mixed-method, single group, pre–post pilot study Level III	30 Latino/a/x families with a child diagnosed with IDD in Texas and Illinois	Trained community healthcare workers with similar cultural backgrounds leading individual and group sessions effectively supports intervention outcomes. Co-development of programs with families and community stakeholders ensured cost-effective, family-centered, culturally relevant care.	Intervention models should emphasize co-creation of services with culturally relevant stakeholders and supports evidence-based practice for community-led models of care.

## Data Availability

No new data were created.
